# Complete Genome Resequencing of Thermus thermophilus Strain TMY by Hybrid Assembly of Long- and Short-Read Sequencing Technologies

**DOI:** 10.1128/MRA.00979-21

**Published:** 2021-11-18

**Authors:** Kentaro Miyazaki, Natsuko Tokito

**Affiliations:** a Bioproduction Research Institute, National Institute of Advanced Industrial Science and Technology (AIST), Tsukuba, Ibaraki, Japan; b Department of Computational Biology and Medical Sciences, Graduate School of Frontier Sciences, The University of Tokyo, Kashiwa, Chiba, Japan; Montana State University

## Abstract

Complete genome resequencing was conducted for Thermus thermophilus strain TMY by hybrid assembly of Oxford Nanopore Technologies long-read and MGI short-read data. Errors in the previously reported genome sequence determined by PacBio technology alone were corrected, allowing for high-quality comparative genomic analysis of closely related T. thermophilus genomes.

## ANNOUNCEMENT

Thermus thermophilus is an aerobic, thermophilic bacterium that grows optimally at around 70 to 75°C. Since the first isolation of this species from Mine Hot Spring in Japan in 1968 (1, 2), many T. thermophilus strains have been isolated from various thermal areas worldwide (3–11). Among them, strains HB8 (type strain) and HB27 have been rigorously studied biochemically (12, 13), structurally (14, 15), and genetically (16, 17).

So far, 13 complete genome sequences have been determined for T. thermophilus strains (https://www.ncbi.nlm.nih.gov/genome/browse#!/prokaryotes/461/). One of the strains, TMY, was isolated from the Otake geothermal power plant in Japan (6), and its genome sequence has been reported previously (18). However, because the sequencing was performed using PacBio technology alone, it contained numerous errors (frameshifted proteins), resulting in exclusion from the RefSeq database. For fine-scale comparative analysis of closely related T. thermophilus genomes, here, we reinvestigated the genome of TMY by combining Oxford Nanopore Technologies (ONT) long-read and MGI short-read sequencing technologies.

Freeze-dried TMY cells (JCM 10668) obtained from JCM (RIKEN, Japan) were inoculated into 5 ml of *Thermus* medium (ATCC 697), containing 0.4 mM MgCl_2_ and 0.35 mM CaCl_2_. After 24 h of cultivation at 70°C, genomic DNA was purified from pelleted cells using a blood and cell culture DNA midi kit (Qiagen). For long-read sequencing, unsheared genomic DNA (1 μg) was pretreated using a short-read eliminator kit (Circulomics) to remove fragments of <10 Kbp, and a library was constructed using a ligation sequencing kit (ONT). Sequencing was performed using a GridION X5 system on a FLO-MIN106 R9.41 flow cell (ONT). Base calling was conducted using Guppy v.4.0.11 to generate 375,245 reads (average, 4,578 bases; total, 1.72 Gb). For all software, default parameters were used. The raw sequencing data were filtered (Q < 10; length, <1,000 bases) using NanoFilt v.2.7.1 (19), yielding 213,707 reads (longest read, 163,227 bases; *N*_50_, 9,231 bases; total, 1.15 Gb). For short-read sequencing, a library was constructed using an MGIEasy FS PCR-free DNA library prep set (MGI) with a ∼400 to 500-bp insert. Paired-end sequencing was then performed on a DNBSEQ-400 instrument (MGI), yielding 8,454,552 paired-end reads (2 × 150 bases). The raw sequencing data were filtered (Q < 30; length, <10 bases) using fastp v.0.20.1 (20), yielding 5,182,387 paired-end reads (average, 150 bases; total, 2.53 Gb). The trimmed long- and short-read data were assembled using Unicycler v.0.4.8 (21), and the assembly was polished using Pilon v.1.23 (22), generating a single circular chromosome and a single circular plasmid. Automatic annotation was conducted using DFAST v.1.4.0 (23), and the genomic features are summarized in Table 1. Comparative sequence analysis between the previously reported sequence (chromosome, GenBank accession number AP017920.1; plasmid pTMY, AP017921.1) (18) and the present result revealed 98.2% pairwise identity. The majority of differences were single-nucleotide deletions in the PacBio sequence, while the presence of two large insertions (∼30 Kbp total) in our sequence was experimentally confirmed by Sanger sequencing.

**TABLE 1:** Genome statistics and features of Thermus thermophilus strain TMY

Genetic element	Length (bp)	GC content (%)	No. of coding DNA sequences	No. of rRNAs	No. of tRNAs	GenBank accession no.
Chromosome	2,151,326	69.0	2,309	6	52	AP025158
Plasmid	19,144	67.4	26	0	1	AP025159

### Data availability.

The complete genome sequence of T. thermophilus TMY is available from DDBJ/EMBL/GenBank under the accession numbers summarized in Table 1. The raw sequencing data were deposited in the SRA database under the accession numbers DRR313875 (Nanopore) and DRR313876 (DNBSEQ).
